# Self-Reported Coffee Consumption and Central and Peripheral Blood Pressure in the Cohort of the Brisighella Heart Study

**DOI:** 10.3390/nu15020312

**Published:** 2023-01-08

**Authors:** Arrigo F. G. Cicero, Federica Fogacci, Sergio D’Addato, Elisa Grandi, Elisabetta Rizzoli, Claudio Borghi, on behalf of the Brisighella Heart Study

**Affiliations:** 1 Hypertension and Atherosclerosis Research Group, Medical and Surgical Sciences Department, Sant’Orsola-Malpighi University Hospital, 40138 Bologna, Italy; 2 IRCCS AOU S. Orsola di Bologna, 40138 Bologna, Italy

**Keywords:** coffee, epidemiology, positive nutrition, blood pressure, central blood pressure, peripheral blood pressure, arterial stiffness

## Abstract

Even though coffee consumption has been clearly related to a number of benefits to the cardiovascular system, its effect on blood pressure (BP) has not been fully elucidated. In this sub-analysis of the Brisighella Heart Study (BHS), we compared central and peripheral BP values in a sub-cohort of 720 men (47.9%) and 783 women (52.1%) reporting the drinking of different amounts of coffee each day, for whom a full set of clinical, laboratory and hemodynamic parameters was available. According to our observations, moderate coffee drinking was associated to either higher levels of systolic BP (SBP) compared to those with heavy coffee consumption or lower SBP than that in the non-coffee drinking group (*p*-value for trend <0.05). In particular, people who drank 2 cups of coffee per day and people who drank >3 cups per day had lower SBP than non-coffee drinkers by 5.2 ± 1.6 mmHg (*p* = 0.010) and 9.7 ± 3.2 mmHg, respectively (*p* = 0.007). Similar trends were also observed for peripheral pulse pressure (PP), aortic BP and aortic PP. In the age-adjusted multiple linear regression model, negative predictors of SBP, PP, aortic BP and aortic PP were the estimated glomerular filtration rate (eGFR), female sex and coffee consumption. Positive predictors included body mass index (BMI) and low-density lipoprotein cholesterol (LDL-C). Then, our findings show that regular coffee drinking is associated with lower SBP, PP, aortic BP and aortic PP, but with similar arterial stiffness.

## 1. Introduction

Coffee is one of the most widely consumed beverages in the world [1]; in 2020/2021, around 166.63 million 60 kg bags of coffee were consumed worldwide. A growing number of epidemiological studies advocates for its consumption for the prevention of cardiovascular disease (CVD), stroke and mortality; a recent large meta-analysis of observational longitudinal studies including more than 12,000,000 participants and registering 36,352 cardiovascular disease cases concluded that there was a non-linear protective association between long-term coffee consumption and cardiovascular events. In detail, compared to that with no coffee consumption, the relative risk of cardiovascular disease is 0.85 (95% confidence interval, 0.80–0.90) for a median 3.5 cups consumed per day [2]. Similar data have been recently confirmed in the US population [3]. However, it is not fully clear if this positive effect of coffee is supported or attenuated by coffee consumption. In fact, the effect of coffee on arterial blood pressure (BP) is still debated, mainly because of the known acute impact of caffeine on BP [4]. In particular, caffeine is well-known to increase BP by interacting with adenosine receptors in arterial vessels [5] and acutely augmenting catecholamine plasma levels [6]. However, this effect is counteracted by antioxidant components in coffee, which cause nitric oxide-mediated vasodilation and downregulate reactive oxygen species levels, finally playing an important role in the regulation of vascular tone [5]. These observations have been recently supported by a systematic review and meta-analysis pooling data from four studies and 196,256 individuals, which found the existence of a non-linear inverse dose–response relationship between coffee consumption and the risk of hypertension development [7]. Therefore, the most recent evidence excludes the significant effect of coffee consumption on BP control in treated and untreated hypertensive individuals [4]. However, to the best of our knowledge, a limited number of studies has investigated the impact of regular coffee intake in both peripheral and central BP parameters, in particular in rural cohorts, where the usual intake of healthy diets rich in bioactive phenols could be more prevalent than that in other more industrialized areas.

In this context, the aim of this analysis was to evaluate the epidemiological association between coffee drinking habits and peripheral and central blood pressure parameters, as well as arterial stiffness parameters, in a well-characterized Italian rural population sample.

## 2. Materials and Methods

### 2.1. The Brisighella Heart Study Design and Participants

The methodology of the Brisighella Heart Study (BHS) has been previously described in detail [8]. In brief, the BHS is the longest-running European epidemiological study, being active since 1972, based on a randomized sample representative of the rural population of Brisighella, a small town in Northern Italy. At the baseline, the BHS involved 2939 adult Caucasian individuals (1491 men and 1448 women) free from CVD. Since then, participants have been clinically evaluated every 4 years, and clinical data and biochemical parameters have been collected each time. Mortality and morbidity data have also been recorded throughout the years, as well as the incidence of the main CV risk factors.

The study was carried out in accordance with the declaration of Helsinki and its amendments. The BHS protocol was approved by the Institutional Ethical Board of the IRCCS Azienda Ospedaliera Universitaria di Bologna (Code: BrixFollow-up_1972-2024). Written informed consent was sought from all study participants.

### 2.2. Clinical and Laboratory Assessments

Quadrennial clinical evaluation included a detailed personal history and physical examination, assessing lifestyle and dietary habits, smoking status and ongoing pharmacological treatments, anthropometric measurements, resting BP, heart rate and a 12-lead electrocardiogram [9]. Family health history was also recorded as a key component of risk assessment for CVD [10].

A validated semi-quantitative questionnaire—the Dietary Quality Index—was used to gather information regarding food intake in the 12 months prior to the visit [11,12]. Data sampled in the BHS with this questionnaire administered by trained personnel have been largely used for previous scientific reporting [10,13]. The questionnaire included a specific question on coffee consumption in terms of number of cups of coffee consumed per day.

Height, weight and waist circumference were measured via standard procedures. Body mass index (BMI) was quantified by dividing weight in kilograms (kg) by height in meters squared (kg/m^2^) and treated as a continuous variable. Systolic (SBP) and diastolic (DBP) BP were measured three times at 1 min intervals, with the patient seated and resting for at least 5 min. As the International guidelines recommend, three BP measurements were averaged and used as the study variable [14]. Pulse pressure (PP) was calculated as the difference between SBP and DBP. Mean arterial pressure (MAP) was calculated as DBP + 1/3 (SBP-DBP). Hypertension was diagnosed as SBP ≥ 140 mmHg and/or DBP ≥ 90 mmHg, or in individuals undergoing treatment with antihypertensive drugs. The central BP, augmentation index (i.e., a measure of systemic arterial stiffness derived from the ascending aortic pressure waveform) and carotid–femoral pulse wave velocity (cfPWV) were noninvasively measured using the Vicorder^®^ apparatus (Skidmore Medical Ltd., Bristol, UK), a validated, commercially available oscillometric device, which guarantees excellent intra- and inter-operator reliability and that is widely used in epidemiological studies [15,16]. Central BP parameters were derived from brachial BP waveforms self-calibrated to brachial SBP and DBP, according to a validated brachial-to-aortic transfer function [17,18].

Participants of the BHS also provided overnight fasting blood samples to measure routine laboratory parameters, such as total cholesterol (TC), triglycerides (TG), high-density lipoprotein cholesterol (HDL-C), creatinine, fasting plasma glucose (FPG) and serum uric acid (SUA). In samples with TG < 400 mg/dL, the Friedewald equation was used to calculate low-density lipoprotein cholesterol (LDL-C). The glomerular filtration rate (eGFR) was estimated based on the Chronic Kidney Disease Epidemiology Collaboration (CKD-EPI) formula. All laboratory analyses were performed with standardized methods by trained personnel [19,20].

### 2.3. Analysis of Hemodynamic Response to Coffee Consumption

**Figure 1:**
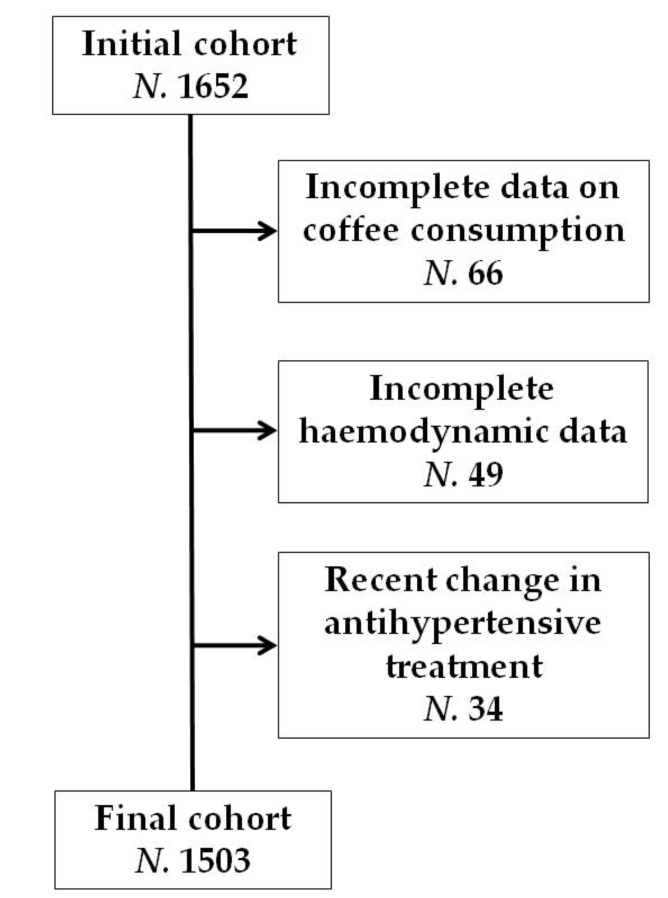
Flow diagram of study population.

During the last BHS population survey, we consecutively evaluated 1652 adult volunteers (Female: 53.4%). From the initial population, 66 participants were excluded due to incomplete information associated with coffee consumption. Individuals with incomplete hemodynamic data (N. 49) were subsequently excluded. Lastly, those who had recently switched antihypertensive medications at the time of the visit (N. 34) were excluded from the analysis (Figure 1).

### 2.4. Statistical Analysis

Continuous variables were presented as the mean and standard deviation (SD). Categorical variables were presented as counts and percentages.

A Kolmogorov–Smirnov normality test was performed for all continuous variables. Characteristics of participants based on either sex or daily coffee intake were compared using a chi-square test for categorical variables and an analysis of variance (ANOVA) test followed by the Tukey post-hoc test for continuous variables. Non-normally distributed parameters were log-transformed before proceeding with the analyses. Spearman’s rank correlation test was carried out on daily coffee intake and other continuous variables. Then, a multiple regression analysis was carried out in order to check the best predictors of SBP, PP, aortic BP and aortic PP in an age-adjusted model, with sex, smoking habits, physical activity, waist circumference, BMI, LDL-C, TG, SUA, eGFR, total fructose load, alcohol and daily coffee intake as predictors. All tests were performed using SPSS 26.0 for Windows (IBM Corporation, Armonk, NY, USA); *p*-values < 0.05 were regarded as statistically significant for each statistical test performed.

## 3. Results

In this analysis, from the BHS, we included 720 men (47.9%) and 783 women (52.1%). Men and women had similar age, LDL-C, TG, FPG, SBP, central BP and arterial stiffness parameters (*p* always > 0.05). The prevalence of hypertension was also similar in men (45.5%) and women (45%) (*p* > 0.05 for the comparison). Women had significantly higher HDL-C, PP and HR and lower SUA and DBP values than men (*p* < 0.05 for the comparisons). The main clinical and laboratory characteristics of the entire cohort and by sex are shown in Table 1.

In the cohort, 220 individuals (14.6%) did not drink coffee regularly, 406 individuals (27%) drank one cup of coffee per day, 726 individuals (48.3%) drank two cups of coffee per day, 99 individuals (6.6%) drank three cups of coffee per day and 52 individuals (3.5%) drank more than three cups of coffee per day (Table 2).

Active smokers were equally distributed among the considered coffee consumer subgroups: non-drinkers, 18%; 1 cup per day drinkers, 17%; 2 cups per day drinkers, 21%; 3 cups per day drinkers, 20%; more than 3 cups per day drinkers, 18% (*p* > 0.05).

There were no significant differences in distribution by sex or prevalence of hypertension among groups according to the number of cups of coffee consumed per day (*p* > 0.05, Table 2). Age, BMI, DBP, MAP, augmentation index, cfPWV, heart rate, plasma lipids, FPG, SUA and creatinine did not significantly differ among the pre-specified groups (*p* > 0.05 for all comparisons). However, a slight trend towards significance was found in eGFR.

**Figure 2:**
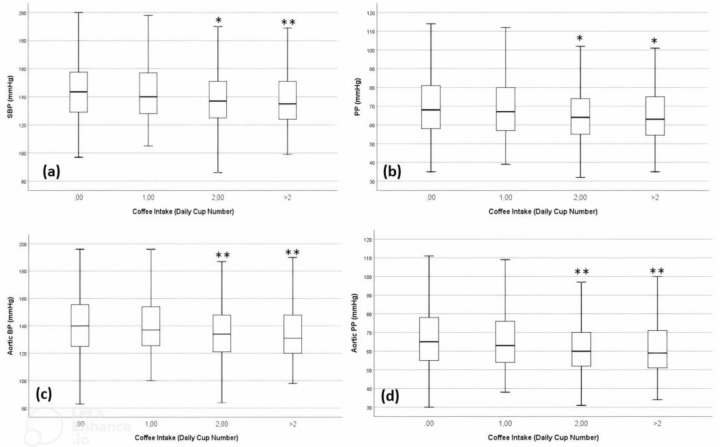
Distribution of (**a**) systolic blood pressure (SBP), (**b**) pulse pressure (PP), (**c**) aortic BP and (**d**) aortic PP in the BHS by coffee consumption (* *p* < 0.05 vs. no coffee intake, ** *p* < 0.05 vs. no coffee and 1 coffee cup intake).

With an increasing number of coffee cups, the SBP tended to decrease (*p*-value for trend tests across the groups < 0.05) (Figure 2a). In particular, people who drank 2 cups of coffee per day and people who drank >3 cups per day had lower SBP by 5.2 ± 1.6 mmHg (*p* = 0.010) and 9.7 ± 3.2 mmHg (*p* = 0.007), respectively, than non-coffee drinkers (Table 2)

Similarly, with an increasing number of coffee cups, peripheral PP (Figure 2b), aortic BP (Figure 2c) and aortic PP (Figure 2c) tended to be lower (*p*-value for trend tests across the groups < 0.05). In particular, people who drank 1 cup of coffee per day showed respective 4.7 ± 1.3 mmHg, 4.9 ± 1.6 mmHg and 4.6 ± 1.3 mmHg decreases in PP (*p* = 0.002), aortic BP (*p* = 0.020) and aortic PP (*p* = 0.020) values than non-coffee drinkers. People drinking more than 3 cups of coffee per day had respective 6.9 ± 2.6 mmHg, 9.5 ± 3.2 mmHg and 6.9 ± 2.6 mmHg decreases in PP (*p* = 0.018), aortic BP (*p* = 0.018) and aortic PP (*p* = 0.018) values than non-coffee drinkers (Table 2). Trends are graphically represented in Figure 2, where the categories 3 and >3 coffee cups/day consumers were grouped in order to compare more balanced groups of subjects (as both 3 and >3 coffee cups/day consumer groups were relatively smaller compared to the previous categories).

In the age-adjusted multiple linear regression model, negative predictors of SBP, peripheral PP, aortic BP and aortic PP were the estimated glomerular filtration rate (eGFR), female sex and coffee consumption. Positive predictors included body mass index (BMI) and low-density lipoprotein cholesterol (LDL-C). More details and coefficients are reported in Table 3.

The results were confirmed after stratifying by the presence of hypertension.

## 4. Discussion

It has been widely demonstrated that lifestyle improvement is the cornerstone of CVD prevention [21]. Among lifestyle strategies, a healthy diet is one of the most effective strategies for attaining BP reduction and control [22]. In effect, a large body of evidence suggests that some food or food components—along with low-dose daily intake of sodium—might provide a specific advantage for BP [23].

Coffee is one of the most widely consumed beverages in Italy and in the world, and its consumption has already been associated with a positive impact on human health, particularly regarding CVD, type 2 diabetes and a number of neurodegenerative and liver diseases [24], even though some subgroups of subjects, whose characteristics have yet to be better defied, could not be protected by coffee intake [2]. In particular, it seems that normoweight and obese patients are less protected than overweight ones [25]. The lack of a difference in the impact of caffeinated and decaffeinated coffee on hard outcomes suggests that caffeine per se is not the main determinant of the effect of coffee on human health [26]. Effectively, caffeine is just one of the several bioactive compounds in coffee, which contains amounts of phenolic compounds (i.e., chlorogenic acids, cafestol, kahweol), alkaloids (trigonelin and, of course, caffeine), diterpenes (i.e., cafestol, kahweol) and other secondary metabolites all potentially involved in a large number of metabolic pathways in humans [27]. Even though caffeine could increase BP levels (especially in individuals who usually do not drink coffee), the amount of coffee bioactive compounds seems to counterbalance this effect with a final neutral-to-positive effect on BP [4]. In particular, chlorogenic acid, which is the most concentrated polyphenol in coffee beans and for which the bioavailability is inversely proportional to the coffee bean roasting time, is thought to be one of the main compounds responsible for the potential BP-lowering effect of coffee [28]. Quercetin, even if characterized by low bioavailability, could also contribute to the positive vascular effect of coffee [29], also acting as a mild xanthine oxidase inhibitor. Moreover, it must be emphasized that coffee consumption is usually safe, even in individuals with high CV risk. In regards to this topic, a meta-analysis of six prospective cohort studies has recently shown that coffee consumption is associated with a lower risk of CV mortality (hazard ratio (HR) = 0.70; 95% confidence interval (CI) 0.54–0.91) as compared to that with no coffee drinking in patients with a previous myocardial infarction [30]. Of course, coffee consumption exceeding the standard could be yet associated with a potential risk of adverse cardiovascular events [2].

In our study, regular self-reported coffee consumption was associated with lower levels of SBP, PP, aortic BP and aortic PP compared to that without coffee consumption. While the association between moderate coffee consumption and lower peripheral BP levels has been clearly observed in a number of large epidemiological studies [4], the observation of coffee consumption with lower central blood pressure has not been as deeply investigated. A large meta-analysis of 24 prospective cohort studies (N. 146,986 individuals) concluded that the adjusted pooled hazard ratio of total cardiovascular events was 1.10 (95% CI 1.04–1.16) for a 10 mmHg increase in aortic BP and 1.12 (95% CI 1.05–1.19) for a 10 mmHg increase in aortic PP. Furthermore, the pooled hazard ratio of all-cause mortality was 1.22 (95% CI 1.14–1.31) for a 10 mmHg increase in aortic PP [31]. Therefore, the epidemiological relevance of our observation about the association between coffee intake and lower central BP could be quite relevant. Moreover, daily coffee intake was also one of the main predictors of BP values in our population sample, together with sex and BMI. Actually, the positive impact of coffee intake on central BP parameters had already been observed in a smaller Swiss cohort [32], but data on larger populations were lacking until now.

Previous analyses carried on the BHS cohort identified fructose load and SUA as predictors of higher cfPWV, but not higher peripheral and central BP [10,18]. In the present study, higher self-reported coffee consumption was found to be associated with lower peripheral and central BP. This observation supports the idea that the interaction between dietary and metabolic factors and BP is complex and that the final effect might be mediated by a number of behavioral variables.

In this study, we did not observe any clinically relevant association between arterial stiffness and self-reported daily coffee consumption, apart from a trend toward a lower augmentation index in mild and strong coffee drinkers compared to that in non-coffee drinkers. This is partially in contrast to what reported in a recent small systematic review of observational studies, showing a negative effect of coffee intake on arterial stiffness [33]. Of course, those findings might be biased by the small number of studies included in the systematic review, as was also acknowledged by the authors.

Of course, our study has some limitations. Firstly, in the BHS, coffee consumption was self-reported, and thus, it may have been underreported. However, trained personnel administered the dietary questionnaire in order to improve the quality of reporting. Secondly, we assessed for coffee consumption regardless of the roasts, origins, or preparation methods and whether it is consumed with milk, with cream or black. Considering that, in Italy, the use of boiled coffee and cream is negligible, and we are confident that the method of coffee consumption in the BHS cohort was overall homogenous. Actually, we were not even able to estimate the consumption of decaffeinated coffee in our sample. However, considering the rural nature of the BHS population, it is possible to state with good approximation that most involved volunteers regularly drink caffeinated coffee. Of course, as stated above, we also think that the positive effect on BP that we recorded in our population sample could be more likely related to coffee bioactive compounds different from caffeine, as also observed in other population studies. Then, we were not able to perform a sub-analysis by sex, because of the risk associated with analyzing very small population subsamples, which could lead to unreliable results. However, most of the studied hemodynamic parameters were balanced between men and women, and between the only two differently distributed parameters, DBP seems to not be related to coffee consumption; moreover, in the regression analysis, sex was not predictive of the different considered BP levels. Another limitation to be acknowledged is that we did not sample urine sodium levels. For this reason, we cannot adjust the hemodynamic response to coffee consumption for dietary sodium intake. However, there is no reason to think that differences in sodium intake exist between the pre-specified groups. Therefore, some of the subjects involved in the study were treated with antihypertensive drugs. To reduce the risk of bias, we excluded the individuals who had recently switched antihypertensive medications at the time of the visit from the analysis. Finally, subjects involved in our cohort had some specific characteristics (rural, with preserved dietary habits during the last decades, with genetic homogeneity, and with a full set of central BP and arterial stiffness data available) that make the obtained data hardly comparable with those obtained in other Italian (and European) cohorts. In particular, a comparison with other European cohorts is difficult because of the different dietary pattern in Northern-Italy compared to that in other countries, including a different method of coffee preparation and consumption and a different amount and biovariability of bioactive peptides and polyphenols included in daily consumed foods. On the other side, our study was one of the first ones to observe a positive association between long-term coffee use and central BP parameters in an Italian rural population sample.

## 5. Conclusions

Based on our observations, self-reported regular coffee drinkers have significantly lower peripheral (SBP, PP) and aortic (central BP and PP) blood pressure than non-coffee drinkers. However, self-reported coffee consumption seems to not be significantly associated with arterial stiffness parameters. Therefore, our data support the overall positive effect of coffee drinking on cardiovascular risk profiles of the general Italian population.

## Figures and Tables

**Table 1:** Main clinical and laboratory characteristics of the entire study population and by sex.

	Clinical and Laboratory Data				Hemodynamic Data		
	Entire Cohort (N. 1503)	Men (N. 720)	Women (N. 783)		Entire Cohort (N. 1503)	Men (N. 720)	Women (N. 783)
Age (years)	58 ± 16	58 ± 15	58 ± 16	SBP (mmHg)	141.0 ± 10.4	140.8 ± 8.5	141.2 ± 12.8
BMI (kg/m^2^)	26.7 ± 4.6	27.1 ± 3.9	26.3 ± 5.1	DBP (mmHg)	73.5 ± 5.7	75.3 ± 5.7	71.9 ± 5.5 *
LDL-C (mg/dL)	140 ± 27	137 ± 26	143 ± 27	MAP (mmHg)	96.1 ± 8.5	97.2 ± 7.5	94.9 ± 8.4
TG (mg/dL)	119 ± 70	126 ± 79	111 ± 60	PP (mmHg)	67.5 ± 6.5	65.4 ± 4.3	69.3 ± 8.7 *
HDL-C (mg/dL)	52 ± 12	48 ± 11	55 ± 12 *	Aortic BP (mmHg)	137.9 ± 10.9	137.5 ± 8.6	138.3 ± 12.8
FPG (mg/dL)	96 ± 19	98 ± 21	93 ± 18	Aortic PP (mmHg)	64.4 ± 7.8	62.1 ± 4.2	66.4 ± 8.6
SUA (mg/dL)	5.3 ± 1.2	5.9 ± 1.1	4.7 ± 1.1 *	Augmentation Index	25.6 ± 6.9	24.3 ± 6.8	26.8 ± 6.9
Creatinine (mg/dL)	1.0 ± 0.2	1.1 ± 0.2	1.0 ± 0.2	cfPWV (m/s)	9.1 ± 2.4	9.2 ± 2.2	9.1 ± 2.5
eGFR (mL/min)	71 ± 16	73 ± 15	69 ± 15	HR (bpm)	64 ± 8	61 ± 7	66 ± 8 *

* *p* < 0.05 versus men. BMI = body mass index, BP = blood pressure, cfPWV = carotid-femoral pulse wave velocity, DBP = diastolic blood pressure, eGFR = estimated glomerular filtration rate, FPG = fasting plasma glucose, HR = heart rate, MAP = mean arterial pressure, N = number of individuals, PP = pulse pressure, SBP = systolic blood pressure, SUA = serum uric acid, TG = triglycerides.

**Table 2:** Main clinical and laboratory characteristics of the study population by cups of coffee consumed per day.

	Clinical and Laboratory Data						Hemodynamic Data				
	Non-Coffee Drinkers (N. 220)	1 Cup of Coffee per Day (N. 406)	2 Cups of Coffee per Day (N. 726)	3 Cups of Coffee per Day (N. 99)	>3 Cups of Coffee per Day (N. 52)		Non-Coffee Drinkers (N. 220)	1 Cup of Coffee per Day (N. 406)	2 Cups of Coffee per Day (N. 726)	3 Cups of Coffee per Day (N. 99)	>3 Cups of Coffee per Day (N. 52)
Age (years)	60 ± 17	58 ± 16	57 ± 15	55 ± 13	55 ± 15	SBP (mmHg)	145 ± 10	144 ± 11	139 ± 10	140 ± 10	136 ± 11 *
BMI (kg/m^2^)	26.7 ± 4.4	26.6 ± 4.4	26.8 ± 4.8	26.9 ± 4.5	26.5 ± 4.2	DBP (mmHg)	74 ± 5	74 ± 10	73 ± 9	75 ± 10	74 ± 10
LDL-C (mg/dL)	139 ± 27	138 ± 26	143 ± 27	138 ± 27	140 ± 36	MAP (mmHg)	97 ± 6	97 ± 6	95 ± 6	96 ± 6	94 ± 7
TG (mg/dL)	119 ± 64	119 ± 76	119 ± 70	113 ± 68	114 ± 66	PP (mmHg)	69 ± 8	70 ± 9	66 ± 8	67 ± 7	62 ± 7 *
HDL-C (mg/dL)	52 ± 12	48 ± 11	51 ± 11	52 ± 10	52 ± 12	Aortic BP (mmHg)	141 ± 10	141 ± 11	136 ± 10	137 ± 11	133 ± 11 *
FPG (mg/dL)	96 ± 13	96 ± 17	95 ± 19	93 ± 13	94 ± 12	Aortic PP (mmHg)	68 ± 4	67 ± 5	63 ± 7	64 ± 8	61 ± 9 *
SUA (mg/dL)	5.3 ± 1.3	5.3 ± 1.4	5.2 ± 1.3	5.2 ± 1.3	5.9 ± 1.4	Augmentation Index	26 ± 4	25 ± 5	25 ± 6	25 ± 7	26 ± 8
Creatinine (mg/dL)	1.0 ± 0.2	1.0 ± 0.2	1.0 ± 0.2	1.0 ± 0.2	1.1 ± 0.2	cfPWV (m/s)	9.4 ± 2.4	9.1 ± 2.7	9.1 ± 2.2	8.8 ± 2.2	9.1 ± 2.6
eGFR (mL/min)	71 ± 16	73 ± 15	69 ± 15	70 ± 16	67 ± 16	HR (bpm)	65 ± 12	64 ± 12	64 ± 11	64 ± 11	64 ± 14

* *p* for trend tests across the groups < 0.05. BMI = body mass index, BP = blood pressure, cfPWV = carotid-femoral pulse wave velocity, DBP = diastolic blood pressure, eGFR = estimated glomerular filtration rate, FPG = fasting plasma glucose, HR = heart rate, MAP = mean arterial pressure, N = number of individuals, PP = pulse pressure, SBP = systolic blood pressure, SUA = serum uric acid, TG = triglycerides.

**Table 3:** Age-adjusted multiple linear regression model identifying the predictors of SBP, PP, aortic BP and aortic PP in the population.

Hemodynamic Parameter	Predictors	B	95% Confidence Interval		*p*-Value
			Lower Limit	Upper Limit	
SBP (mmHg)	eGFR (ml/min/1.73 m^2^)	−0.306	−0.351	−0.261	<0.001
	BMI (kg/m^2^)	1.461	1.236	1.686	<0.001
	Coffee Consumption	−1.734	−2.876	−0.593	0.003
	LDL-C (mg/dL)	0.039	0.012	0.065	0.004
	Sex (M versus F)	−2.716	−4.757	−0.676	0.009
PP (mmHg)	eGFR (ml/min)	−0.263	−0.298	−0.228	<0.001
	BMI (kg/m^2^)	1.027	0.845	1.208	<0.001
	Coffee Consumption	−1.544	−2.462	−0.625	0.001
	LDL-C (mg/dL)	0.028	0.007	0.050	0.010
Aortic BP (mmHg)	eGFR (mL/min/1.73 m^2^)	−0.326	−0.371	−0.281	<0.001
	BMI (kg/m^2^)	1.512	1.287	1.738	<0.001
	LDL-C (mg/dL)	0.043	0.016	0.070	0.002
	Coffee Consumption	−1.643	−2.791	−0.495	0.005
	Sex (M versus F)	−2.714	−4.769	−0.659	0.010
Aortic PP (mmHg)	eGFR (mL/min/1.73 m^2^)	−0.283	−0.318	−0.248	<0.001
	BMI (kg/m^2^)	1.078	0.898	1.258	<0.001
	Coffee Consumption	−1.478	−2.391	−0.565	0.002
	LDL-C (mg/dL)	0.032	0.010	0.053	0.004

BMI = body mass index, BP = blood pressure, eGFR = estimated glomerular filtration rate, LDL-C = low-density lipoprotein cholesterol, PP = pulse pressure, SBP = systolic blood pressure.

## Data Availability

The data that support the findings of this study are available from the University of Bologna. Data are available from the corresponding author with the permission of the University of Bologna.
